# Characterization of Treatment Resistant Depression Episodes in a Cohort of Patients from a US Commercial Claims Database

**DOI:** 10.1371/journal.pone.0076882

**Published:** 2013-10-18

**Authors:** Nicole Kubitz, Maneesha Mehra, Ravi C. Potluri, Nitesh Garg, Nicole Cossrow

**Affiliations:** 1 Janssen-Cilag GmbH, Market Access, Global Commercial Strategy Organization, Neuroscience, Janssen-Cilag GmbH, Neuss, Germany; 2 Janssen Global Services, LLC, Market Access Analytics, Global Market Access & Commercial Strategy Organization, Raritan, New Jersey, United States of America; 3 SmartAnalyst Inc, New York, New York, United States of America; University of Glasgow, United Kingdom

## Abstract

**Context:**

Treatment Resistant Depression (TRD) is a significant and burdensome health concern.

**Objective:**

To characterize, compare and understand the difference between TRD and non-TRD patients and episodes in respect of their episode duration, treatment patterns and healthcare resource utilization.

**Design and Setting:**

Patients between 18 and 64 years with a new diagnosis of major depressive disorder (MDD) and without a previous or comorbid diagnosis of schizophrenia or bipolar disease were included from PharMetrics Integrated Database, a claims database of commercial insurers in the US. Episodes of these patients in which there were at least two distinct failed regimens involving antidepressants and antipsychotics were classified as TRD.

**Patients:**

82,742 MDD patients were included in the analysis; of these patients, 125,172 episodes were identified (47,654 of these were drug-treated episodes).

**Main Outcome Measures:**

Comparison between TRD and non-TRD episodes in terms of their duration, number and duration of lines of treatment, comorbidities, and medical resource utilization.

**Results:**

Of the treated episodes, 6.6% (N = 3,134) met the criteria for TRD. The median time to an episode becoming TRD was approximately one year. The mean duration of a TRD episode was 1,004 days (vs. 452 days for a non-TRD episode). More than 75% of TRD episodes had at least four lines of therapy; half of the treatment regimens included a combination of drugs. Average hospitalization costs were higher for TRD than non-TRD episodes: $6,464 vs. $1,734, as were all other health care utilization costs.

**Conclusions:**

While this study was limited to relatively young and commercially covered patients, used a rigorous definition of TRD and did not analyze for cause or consequence, the results highlight high unmet medical need and burden of TRD on patients and health care resources.

## Introduction

Major Depressive Disorder (MDD) is a chronic mood disorder prevalent across the globe. Among adults in ten high-income countries, average lifetime and 12-month prevalence estimates of MDD were 14.6% and 5.5%, respectively (US, 19.2% and 8.3% respectively) and in eight low- to middle-income countries 11.1% and 5.9%, respectively [1]. In the Organization for Economic Cooperation and Development (OECD) Expert Meeting in April 2010, the 12-month prevalence of MDD in the US, Australia and the European Union was reported to be 6.7%, 6.3% and 6.9%, respectively [2].

MDD is more common among women than men and often begins in young adulthood [3] **.** Occurrence of MDD has been reported to be higher or more aggravated in patients suffering from cardiovascular diseases, AIDS, cancer, alcohol dependence and several neurological conditions [3], [4]. Furthermore, many studies have conversely shown depression to be a risk factor for cardiovascular diseases (CVD) along with its associated morbidity, and Type 2 diabetes [5], [6]. Common co-morbidities also associated with MDD include anxiety disorders, chronic pain, osteoarthritis and alcohol dependence [4], [7].

In 2000, MDD was estimated to represent 11% of disabilities from all causes [8]. According to the 2004 WHO report, prevalence of depression is the third highest globally among all disabling conditions of moderate and severe disability [9]. The Global Burden of Disease Study 2010 has ranked major depressive disorder at the 11^th^ rank in 2010, in terms of diseases associated with the most disability-adjusted life years – up from 15^th^ rank in 1990. In addition to the clinical burden of MDD, there is considerable economic burden. In the US alone, the estimated direct and indirect cost amounted to $83 billion in 2000 [10], of which more than 50% of health expenditures were borne by the public sectors such as Medicaid, Medicare, state and local governments [11].

A subset of the MDD population has Treatment Resistant Depression (TRD) which is characterized as MDD that persists even after adequate antidepressant therapy. There is, however, a lack of consensus in defining TRD. The European Union’s Committee for Human Proprietary Medicinal Products (CHMP)’s definition [12] states: ‘a patient is considered therapy-resistant (TRD) when consecutive treatments with two different antidepressant products, used for a sufficient length of time at an adequate dose with adequate affirmation of treatment adherence, fail to induce a clinically meaningful improvement’.

Other definitions of TRD have variously been based on a single criterion such as failure, variably defined on lack of response to antidepressants or on need for use of electroconvulsive therapy (ECT), or on a scoring matrix based on multiple criteria such as number of switches, number of titrations, and use of ECT [8], [13]–[15] **.** This inconsistency in defining TRD across clinical studies has resulted in a wide variation in the estimates of TRD rates. For example, *Souery et al* [16] reported 10%–20% of MDD patients as TRD, *Corey-Lisle et al* determined TRD-likely to be 12% [8] while *Gibson et al* [15] reported this prevalence to be 29%.

The clinical and economic burden seen in MDD is amplified in TRD. Among patients with MDD, TRD patients experience more comorbidities than non-TRD patients. In a comparative study of *likely*-TRD and *non*-TRD employees with MDD, *Ivanova et al* [7] reported prevalence of comorbidities in these two groups: anxiety disorders, chronic pain and fibromyalgia were present in 20.5%, 23.2% and 6.4% of those with TRD vs.12.6%, 14.5% and 3.0% without TRD. TRD is also associated with higher costs relative to non-TRD episodes. In a study using a claims database, the cost of medical services linked with TRD was reported to be more than twice that of non-TRD patients ($10,954 vs. $5,025) [8]. *Crown et al* [17] reported that among patients with MDD, those with TRD were twice more likely to be hospitalized than non–TRD patients, and that health care costs for hospitalized TRD patients were six times more than for non-TRD patients.

The objectives of this study were to identify and characterize patients and their episodes of TRD in a representative sample of commercially insured US patients diagnosed with MDD. Duration and treatment choices of TRD and non-TRD episodes were compared. Within treatment choices, number and duration of lines of treatment, use of monotherapy vs. combination therapy, switching of regimens from one line of treatment to the next, usage of drug classes and drugs prescribed in each pool (TRD and non-TRD) were analyzed. The extent of co-morbidities between these two patient sets was determined. Finally, medical resource utilization (MRU) cost per episode for each pool was analyzed to understand the economic burden of TRD episodes vs. non-TRD episodes.

MDD as well as TRD occur in recurrent episodes [18]; therefore, the analysis for this study was conducted at the episode and not patient level. This afforded the ability to examine and characterize the sequence of drug regimens, lines of therapy and MRU associated with an episode.

## Methods

### Data Source

Anonymized patient longitudinal data were sourced from PharMetrics Integrated Database, a claims database of commercial insurers in the US. The database includes inpatient and outpatient claims, diagnoses and procedures based on ICD-9-CM and CPT-4 codes, as well as retail and mail order pharmacy claims for more than 70 million members from more than 100 health plans across the US. Enrolment data include information on age, gender, and periods of service eligibility. Prescription claims include the National Drug Code, quantity of units dispensed and days of supply. Included data ranged from 1995 to 2010 in order to maximize the continuous evaluation for each patient, though more than 85% of data evaluated were for the period from 2003 to 2010.

### Study Sample

The study sample began with identifying patients with MDD and included 572,682 patients with at least one service claim for a depressive disorder including dysthymic disorder (ICD-9-CM codes 296.2, 296.3, 300.4, 309.1, 311). Patients were excluded if a) they had a pre-defined exclusion diagnosis for schizophrenia, schizoaffective or bipolar disorders (ICD-9-CM codes 295, 296.0, 296.1, 296.4, 296.5, 296.6, 296.7, 296.8, 296.9, 298; b) the patient’s age on Index Diagnosis Date was not within the 18–64 year age bracket; or c) an Index Diagnosis Date was not found. The Index Diagnosis Date for each patient was defined as the date of the first inclusion diagnosis that: 1) had no other inclusion diagnosis or prescription claim of antidepressants in the prior 120 days (guided by the definition of a “new episode” contained in PQRI 2007 Measure 9) [19], and 2) had continuous service eligibility for 4 months prior to and 24 months after the date (to ensure a minimum study period of 2 years for each patient).

### Classification of MDD Episode

After patients were included in the study sample, the data were examined and episodes of MDD were established for each patient. An MDD episode was defined to begin on a first relevant date and end 120 days after the last relevant date, where the first relevant date was a date of inclusion diagnosis that was not preceded by any inclusion diagnosis or an antidepressant prescription claim in the preceding 120 days. The last relevant date was a date of inclusion diagnosis or antidepressant prescription claim that was followed by a clear period of 120 days without any inclusion diagnosis or antidepressant claim.

### Classification of a Regimen

A regimen was defined as all antidepressant and antipsychotic/antimanic drugs (ADAP) that were valid concurrently during an MDD episode. A drug was considered valid during any period if its claim was during that period or if the days’ supply of the claim extended into that period. If only one drug was valid during a period, it was considered a monotherapy regimen of that drug.

Any regimen, once established, was deemed to continue for at least 30 days before any change was considered. Once a regimen began, the next date on which it was checked to see if the regimen changed was the next prescription date after 30 days elapsed. In the interim period, any other drugs prescribed were considered as having been prescribed in combination with the other drugs prescribed during this period. The decision to use 30 days was based on the observation that the days’ supply in more than 80% of prescription claims was 30 days. This identification of regimens was done at the level of individual medications and not drug classes. Thus, a change from one drug to another, even if both drugs were of the same drug class, was treated as a change of regimen. This approach was used because it has been reported that there is insufficient evidence that between-class switching increases the likelihood of achieving either response or remission compared to within-class switching [20]–[22]. A change in regimen was considered a switch to the next line of treatment (LOT).

Each drug was allowed a grace period of 60 days at the end of its validity period to check if it was renewed. If it was not renewed within the grace period, the original validity period stayed unchanged. If it was renewed within the grace period, the validity period was deemed to have extended all the way through the period where it did not have validity. At the expiration of a regimen, if the next regimen commenced within the 30 days, the previous regimen was deemed to extend up to the commencement of the new regimen. If no regimen commenced within the 30-day period, the previous regimen was deemed to end 30 days after its validity, and a ‘blank’ regimen with no drug therapy, started from that date until the commencement of the next regimen.

### Treatment Resistant Depression (TRD) Episode

An episode was classified as TRD if the episode contained at least 2 distinct failed regimens; failure was based on treatment switch or discontinuation. All regimens in an episode were considered failures, except when a) a regimen was the last regimen of the episode, and b) a regimen had a succeeding step-down regimen (one or more drug(s) was discontinued and no other drug was added), in all instances in which the regimen appeared. The discontinuation of a regimen and not an individual product was considered a failure given the widespread usage of combination therapy particularly in later lines of treatment of MDD.

Only drugs belonging to antidepressant and antipsychotic/antimanic classes were included when determining regimens, not only for the purpose of categorizing episodes as TRD but also for duration and switch analysis. Dosage was not considered in determining change of regimens as it was assumed the physician switched a regimen only after either the dosage of the current regimen was titrated to an appropriate level, or where the change had to be made on account of adverse events even if the recommended or optimal dosage was not reached.

### Comparison of TRD Episodes with Non-TRD Episodes that Contained at Least One ADAP Claim

To carry out one of the main objectives of this study of comparing TRD episodes with non-TRD episodes, the latter were limited to those that had at least one ADAP claim (N = 47,654; 38%). Since the analysis was principally focused on drugs used in treatment of MDD and TRD, the episodes not containing any prescription claims were excluded for this purpose. This is in line with eligibility considered by other studies. *Gibson et al* [15] included patients if they had at least 2 diagnoses claims and 1 antidepressant prescription during the study period. *Crown et al* [17] included for comparison with TRD patients, such patients that were “diagnosed with depression who met initial antidepressant medication dosage selection criteria but did not meet criteria for treatment resistance”.

### Comorbidities and Medical Resource Utilization (MRU)

Comorbidities, specifically in the areas of drug/substance abuse, anxiety, pain, sleep disorders and impaired cognitive functioning were analyzed at the patient level to determine if patients with TRD had a greater occurrence of these comorbidities. Analyses were also conducted at the episode level to examine MRU, including hospitalizations, office visits and prescription filling, to determine whether TRD episodes compared to non-TRD episodes were associated with greater health care utilization and costs.

### Statistical Analysis

Independent samples t tests and large sample tests were used to analyze differences in parameters for TRD and non-TRD patients and episodes. The level of significance for these was fixed at 5%. For t tests, in case of equal variances of the two samples being compared, the pooled method was used to calculate the degrees of freedom, and in case of unequal variances, Satterthwaite’s approximation was used to calculate the degrees of freedom.

The two-tailed t test for difference of means was used to compare patient age, duration of an episode, duration of each line of treatment, cost per episode and rate of visits across different MRU categories for the TRD and non-TRD episodes. The large sample difference of proportions test was used to compare the proportion of female patients, extent of prescription of various AD classes in TRD and non-TRD episodes and the usage of monotherapy and combination regimens in the two cases. The Bonferroni correction was applied to adjust for multiple comparisons, and only values less than 0.00114 (P<0.05/n, n = 44) were considered statistically significant.

## Results

### MDD Patients and Episodes

**Figure 1:**
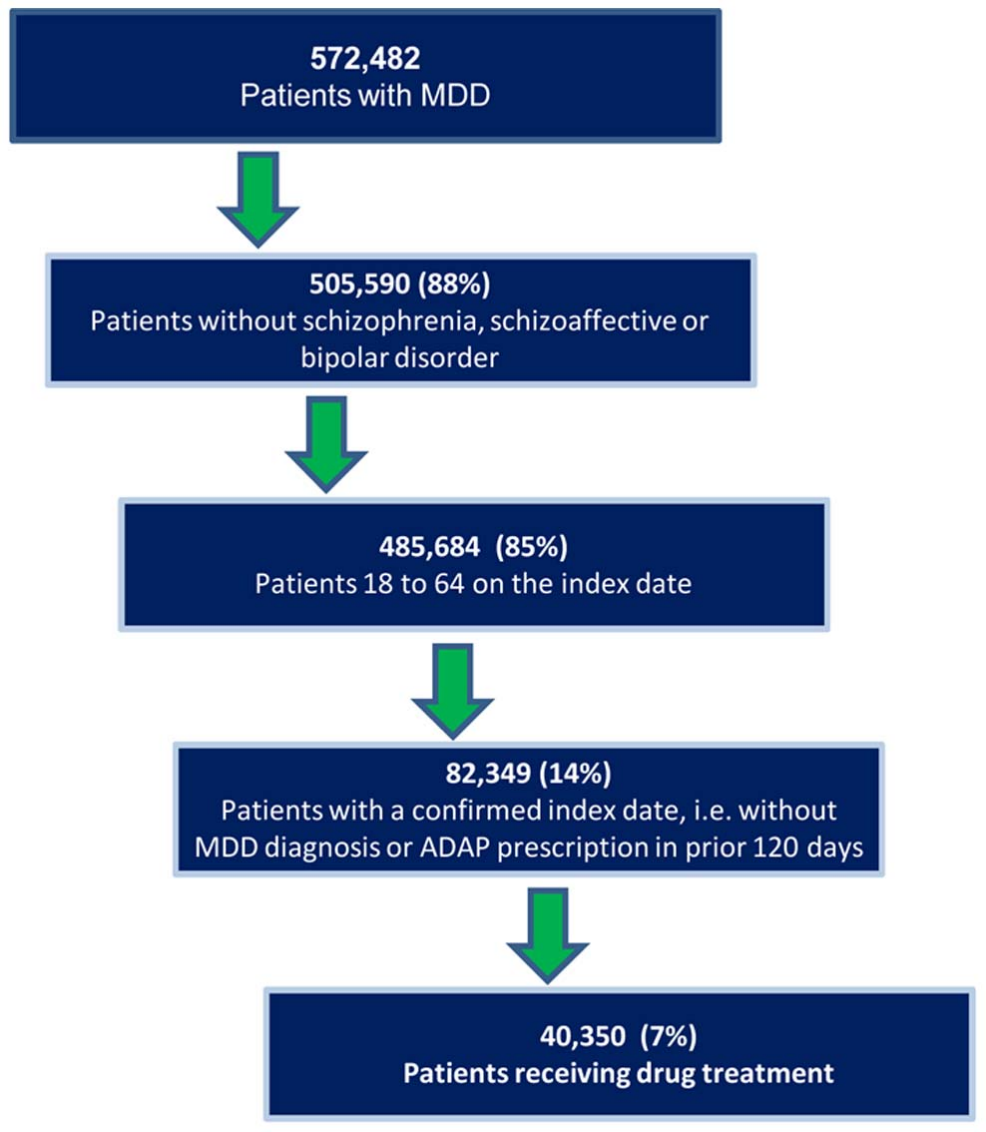
Patient selection flow. Cascade depicting patient selection after applying various filters, and the number of patients excluded at each filter.

The study sample began with 572,482 MDD patients. From those, 66,890 were excluded because they had a diagnosis for schizophrenia, schizoaffective or bipolar disorders; 20,052 patients were excluded because they were not between the ages of 18 and 64 years on their index diagnosis date; 402,796 patients were excluded because an index diagnosis date could not be confirmed (see Figure 1).

In all, 125,172 episodes were identified for the 82,741 patients contained in the sample. Of the patients who met the inclusion criteria for MDD, 67.9% (N = 56,176) were women, and 71.0% were aged between 36 and 64 years. Of the 125,172 episodes, 58% were initially diagnosed with ‘depressive disorder not classified elsewhere’, 24% with MDD – single or recurrent episode, and 17% with dysthymic disorder. Notably, 42% of the episodes were characterized by only the index diagnosis, without any subsequent MDD diagnosis or antidepressant prescription. A further 20% of episodes did not contain any antidepressant prescription while containing a subsequent inclusion diagnosis. Thus, a total of 62% of all episodes had no antidepressant prescription.

### Comparison of TRD Episodes with Non-TRD Episodes that Contained at Least One ADAP Claim

Of the 125,172 identified MDD episodes, 47,654 (38.1%) had an ADAP prescription and were included in the analysis, while 6.6% (N = 3,134) qualified as TRD episodes. If a patient had at least one TRD episode, the patient was classified as a TRD patient. There was no significant difference in age between TRD and non-TRD patients (p = 0.37). TRD was more common among women than men (6.8% vs. 6.0%), leading to a greater proportion of female patients in the TRD group than in the non-TRD group (Table 1). The median duration of TRD episodes (891 days) was more than twice as long as non-TRD episodes (298 days) (p<0.001) and the median time it took for an episode to become TRD was more than one year (Table 1).

**Table 1:** TRD and Non-TRD episode characteristics.

TRD patient characteristics	TRD Episodes	Non-TRD Episodes	p-value
N	3,134	44,520	
Number of patients	3,095	37,255	
Female, N (%)	2,237 (72.3%)	25,733 (69.3%)	<0.001
18–35 years, N (%)	927 (30.0%)	11,397 (30.6%)	0.46
36–50 years, N (%)	1,320 (42.6%)	15,087 (40.5%)	0.02
51–64 years, N (%)	848 (27.4%)	10,771 (28.9%)	0.07
Median Duration	891	298	<0.001
Mean Duration	1,004	452	<0.001
% TRD episodes^a^	6.6%		
% TRD prevalence^b^ (i.e. %TRD episodes, adjusted for duration)	13.6%		
Number of days to become TRD, mean [median], (quartile range)	479, [372],(214–640)	NA	
% of episodes that took more than 360 days to become TRD	52%	NA	

a % TRD episodes are an approximation of TRD incidence.

b TRD prevalence = TRD episode-days/(TRD episode days+non-TRD episode days)x100, where episode days are calculated as the number of episodes multiplied by mean duration.

### Comparison of Treatment Patterns in TRD and Non-TRD Episodes

**Figure 2:**
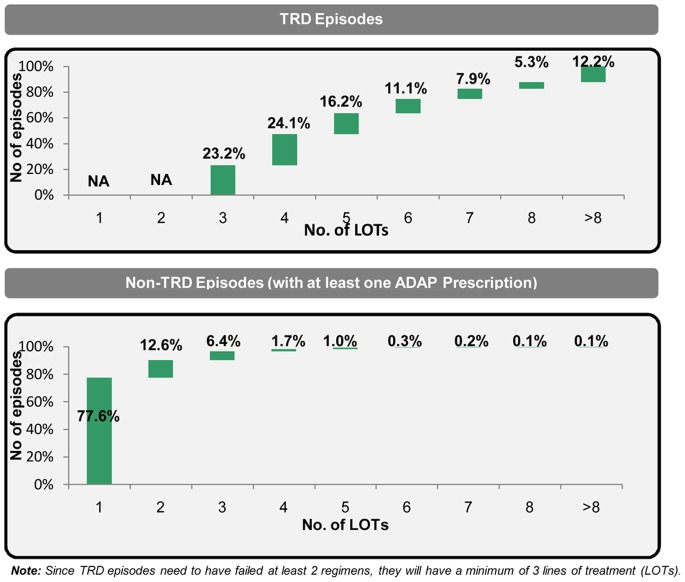
Distribution of episodes based on lines of therapy. Distribution of episodes by number of lines of treatment contained therein, compared for TRD and non-TRD episodes.

More than 75% of TRD episodes had at least four lines of therapy (LOTs) whereas for non-TRD episodes, more than 75% had only one LOT (Figure 2). While TRD episodes were longer, the duration of a LOT till LOT5 was noticeably and statistically significantly shorter in TRD episodes (Table 2). Among the regimens used in TRD episodes, nearly half included a combination of ADAP treatments, whereas for non-TRD, more than 80% of the regimens were monotherapy. Further, the mean duration of a monotherapy regimen was longer than that of combination regimens in both TRD and non-TRD episodes.

**Table 2:** Duration of each line of treatment.

	TRD episodes^a^(N = 3,134)			Non-TRD episodes (with at least one ADAP prescription)(N = 44,520)			p (H_o:_ M_T = _M_N_; H_1:_ M_T_<>M_N_)
Line of Treatment	N	Median duration(In days)	Mean duration(In days)	N	Median duration(In days)	Mean duration(In days)	Comparing Mean Duration(In days)
LOT 1	3,134	93 (55–223)	183	44,520	114 (38–322)	257	<0.001
LOT 2	3,134	69 (48–147)	139	9,952	83 (42–204)	192	<0.001
LOT 3	3,134	72 (45–183)	157	4,341	141 (62–348)	267	<0.001
LOT 4	2,406	84 (47–196)	171	1,512	98 (53–252)	210	<0.001
LOT 5	1,651	85 (49–190)	167	763	114 (56–275)	222	<0.001
LOT 6	1,142	85 (49–165)	154	333	95 (59–228)	188	0.01
LOT 7	794	83 (46–166)	161	185	99 (56–220)	194	0.04
LOT 8	545	87 (45–191)	157	99	106 (49–251)	201	0.05
LOT 9	379	79 (45–170)	144	49	91 (59–148)	154	0.71
LOT 10	264	79 (46–151)	151	26	79 (51–237)	184	0.48

Figures in brackets represent lower and upper quartile limits.

Abbreviations: ADAP: Antidepressant and antipsychotic/antimanic drugs; p = p-value; H_0_ = Null Hypothesis, H_1_ = Alternative Hypothesis; M_T_ = the average duration of a LOT in TRD episodes, M_N_ = the average duration of a LOT in non-TRD episodes; LOT = Line of therapy.

a An episode will have at least 3 lines of treatment to qualify as TRD, based on the definition stated in the Methods section.

### Drug Usage and Switch

Among the drug classes, SSRI (selective serotonin reuptake inhibitor) was the most used class of drugs for both TRD and non-TRD episodes comprising more than 60% of episodes adjusted for duration (69.6% in non-TRD, and 61.6% in TRD). Usage of all other drug classes such as atypical antidepressants (chiefly bupropion), SNRIs (serotonin norepinephrine reuptake inhibitors) and antipsychotics were more prevalent in TRD than non-TRD episodes (Table 3). Bupropion, sertraline, escitalopram, fluoxetine and citalopram were the most commonly used drugs and were used almost equally in both TRD and non-TRD episodes when used as monotherapy. Bupropion dominated combination usage in both TRD and non-TRD episodes; it was contained in each of the top four combinations – with escitalopram, citalopram, fluoxetine and sertraline. Tables 4 and 5 contain the share of various regimens by line of treatment and provide a comparison of the use of regimens in TRD and non-TRD episodes. While use of antipsychotics was relatively uncommon, they were most often used in combination with SSRIs (Tables 4 and 5) followed by their use in combination with SNRIs.

**Table 3:** Use of antidepressant classes as a % of episode duration.

Antidepressant Classes	TRD episodes	Non-TRD episodes (with at leastone ADAP prescription)	P
			H_o:_ M_T = _M_N_; H_1:_ M_T_<>M_N_
SSRI	62%	70%	a<0.001
ATYPICAL AD	24%	16%	<0.001
SNRI	25%	15%	<0.001
AD - OTHERS	24%	8%	<0.001
AP	7%	2%	<0.001
LITHIUM	0.4%	0.0%	<0.001
AD – MAOI	0%	0%	<0.001

Abbreviations: p = p-value, H_0_ = Null Hypothesis, H_1_ = Alternative Hypothesis; M_T_: The proportion of usage of an antidepressant in TRD episodes; M_N_: The proportion of usage of an antidepressant in non-TRD episodes; ADAP: Antidepressant and antipsychotic/antimanic drugs; SSRI: Selective serotonin reuptake inhibitor; SNRI: Serotonin Norepinephrine reuptake inhibitors; AD: Antidepressant; AP: Antipsychotics; MAOI: Monoamine oxidase inhibitors;

a H_1_: M_T_<> M_N._

**Table 4:** Regimen usage by line of treatment in TRD episodes.

	Line of treatment									
Regimen	1	2	3	4	5	6	7	8	9	10
SSRI only	54%	29%	28%	26%	23%	19%	20%	18%	17%	16%
SNRI only	11%	9%	11%	11%	11%	10%	11%	9%	9%	11%
SSRI & Other ADs	7%	12%	9%	10%	10%	12%	9%	11%	11%	10%
SSRI & Atypical ADs	3%	13%	9%	10%	9%	10%	9%	9%	9%	6%
Atypical ADs only	10%	6%	8%	6%	6%	7%	5%	6%	4%	5%
AD – Others only	7%	6%	6%	6%	7%	6%	9%	6%	8%	8%
SNRI & Other ADs	1%	3%	3%	4%	5%	4%	6%	6%	7%	6%
SSRI & SNRI	1%	5%	5%	4%	4%	4%	3%	3%	2%	3%
SSRI & AP	2%	3%	3%	3%	4%	3%	4%	3%	2%	4%
N (Episodes)	3,134	3,134	3,134	2,406	1,651	1,142	794	545	379	264

Note: ‘AD – Others’ includes alpha-2 receptor antagonists (tetracyclics), modified cyclics, tricyclic agents; Atypical AD includes miscellaneous antidepressants, chiefly bupropion;

AP (Antipsychotics) includes benzisoxazoles, butyrophenones, dibenzapines, dihydroindolones, phenothiazines, quinolinone derivatives, thioxanthenes.

Abbreviations: SSRI: Selective serotonin reuptake inhibitor;AD: Antidepressant; SNRI: Serotonin norepinephrine reuptake inhibitors.

**Table 5:** Regimen usage by line of treatment in non-TRD episodes.

	Line of treatment									
Regimen	1	2	3	4	5	6	7	8	9	10
SSRI only	64%	35%	49%	29%	40%	27%	35%	32%	31%	38%
Atypical ADs only	12%	8%	12%	9%	12%	7%	12%	12%	12%	8%
SNRI only	10%	9%	13%	8%	12%	9%	11%	3%	14%	8%
AD–Others only	5%	6%	6%	7%	6%	7%	8%	12%	6%	12%
SSRI & Other ADs	3%	10%	6%	13%	10%	17%	14%	11%	8%	15%
SSRI & Atypical AD	2%	12%	4%	13%	5%	12%	6%	4%	8%	
SSRI & SNRI	0%	4%	1%	3%	2%	3%	1%	2%		
SSRI & AP	1%	3%	1%	4%	1%	4%	3%	5%	6%	8%
N (Episodes)	44,520	9,952	4,341	1,512	763	333	185	99	49	26

Note: ‘AD – Others’ includes alpha-2 receptor antagonists (tetracyclics), modified cyclics, tricyclic agents; Atypical AD includes miscellaneous antidepressants, chiefly bupropion;

AP (Antipsychotics) includes benzisoxazoles, butyrophenones, dibenzapines, dihydroindolones, phenothiazines, quinolinone derivatives, thioxanthenes.

Abbreviations: SSRI: Selective serotonin reuptake inhibitor;AD: Antidepressant; SNRI: Serotonin norepinephrine reuptake inhibitors.

### Comorbidity Analysis

A greater proportion of patients experiencing TRD episodes suffered from the examined co-morbidities significantly more than patients experiencing non-TRD episodes (Table 6). In addition, more than 30% of the TRD patients experienced each of the following conditions: Muscle & Joint Pain, Anxiety & Panic Disorder, Fatigue, Headache/Migraine, while in the case of non-TRD patients, only Muscle & Joint Pain was experienced by more than 30% patients (Table 6). These comorbid conditions were all more prevalent in women than men, both within TRD and non-TRD patients.

**Table 6:** Patients suffering from comorbidities.

Comorbidity	% of TRD patients^a^ withco-morbidity	% of non-TRD patients^b^with co-morbidity	p
			H_o_: C_T = _C_N_; H_1_: C_T_ <>C_N_
Muscle & joint pain	61%	36%	<0.001
Anxiety & panic disorder	50%	29%	<0.001
Fatigue	43%	23%	<0.001
Headache/migraine	35%	17%	<0.001
Sleep disorder	34%	17%	<0.001
Back pain	25%	12%	<0.001
Obesity, weight gain	19%	10%	<0.001

Abbreviations: p = p-value, H**_0_** = Null Hypothesis, H**_1_** = Alternative Hypothesis;

a Distinct patients out of all TRD episodes (with at least one ADAP prescription).

b Distinct patients with all non-TRD episodes.

c C_T_: The proportion of TRD patients suffering from a co-morbidity C_N_: The proportion of non-TRD patients suffering from a co-morbidity.

### Consumption of Medical Resources

Each category of MRU, as shown in Table 7, was statistically significantly greater for TRD than non-TRD episodes. This was driven not just by longer episode duration but also by greater frequency of usage (measured in terms of number of visits per 100 episode days), and higher cost per visit/claim. The combined impact of these factors resulted in TRD episode costs being 2.7–5.8 times higher than the corresponding costs for a non-TRD episode (Table 7).

**Table 7:** Medical Resource Utilization (MRU) – TRD and non-TRD.

MRU type	Duration of episode (days)	aRate of visitsper 100 days	Cost^b^ pervisit ($)	cCost perepisode ($)	TRD costas a multiple of non-TRD cost
		H_o_: M_T_ = M_N_; H_1_: M_T_<> M_N_		H_o_: C_T_ = C_N_; H_1_:C_T_<> C_N_	
	A	B	C	Product of A,B,C	
Hospitalization^d^	1004,452	(0.06,0.04,p<0.001)	(11569,11285)	(6464,1734,p<0.001)	3.7
Pharmacy	1004,452	(7.48,5.15, p<0.001)	(96,89)	(7175,2073,p<0.001)	3.5
Office visits - GP/FP	1004,452	(0.81,0.71, p<0.001)	(60,58)	(489,184, p<0.001)	2. 7
Office visits - Psychiatrist	1004,452	(0.73,0.33, p<0.001)	(73,70)	(534,103, p<0.001)	5.2
ER	1004,452	(0.13,0.09, p<0.001)	(578,346)	(764,131, p<0.001)	5.8
Lab	1004,452	(0.87,0.71, p<0.001)	(73,68)	(641,214, p<0.001)	3.0
Other outpatient^e^	1004,452	(2.04,1.66, p<0.001)	(343,310)	(7048,2327, p<0.001)	3.0

The format for the values is TRD, non-TRD in columns A and C, and (TRD, non-TRD, P-value) in columns B and D.

a M_T_: The average rate of visits per 100 days in TRD episodes; M_N_: The average rate of visits per 100 days in non-TRD episodes.

b Cost is the amount paid by the insurer – appears as paid field in PharMetrics database.

c C_T_: The average cost per TRD episode; C_N_: The average cost per non-TRD episode.

d Based on inpatient costs in PharMetrics; does not include inpatient – SNF costs.

e Other outpatient claims are mainly comprised of devices (orthopedic, catheters, infusion pumps, hearing devices etc), diagnostics, dental procedures.

Abbreviations: MRU: Medical resource utilization; ER: Emergency Room visits; GP: General practitioner; FP: Family practitioner.

H_0_ = Null Hypothesis, H_1_ = Alternative Hypothesis.

An additional analysis revealed that patients had an average of 16 psychotherapy sessions in each TRD episode as compared to only 4 sessions in a non-TRD episode.

## Discussion

MDD is a debilitating disease that affects millions of individuals worldwide. While there is considerable research on patients suffering from depression and treatment-resistant depression, there is not as much research and knowledge centred on episodes of treatment resistant depression. This analysis of a claims database offers insights at both the patient and the episode levels.

Guidelines from the American Psychiatric Association recommend that treatment of MDD to return the patient to a baseline level of function be done with pharmacotherapy, psychotherapy, a combination of the two or some other form of somatic therapy [23]. This study showed that a large percentage (62%) of MDD-diagnosed new episodes is not treated with pharmacotherapy, i.e. treated with neither an antidepressant nor an antianxiety agent, anticonvulsant, antipsychotic/antimanic or hypnotic; psychotherapy and other somatic therapies were not considered for this purpose. This proportion of untreated patients is similar to MDD epidemiology findings from the National Comorbidity Survey [24] –48% of those patients in a current episode of MDD were not receiving health care treatment for their depression. Consequently, there are a number of people with symptoms commonly associated with depression or diagnosed as suffering from MDD, who do not receive depression-related drug or other forms of treatment [24].

The estimate of TRD incidence, based on the percentage of TRD episodes, was 6.6%. This incidence was adjusted for mean episode duration, resulting in an approximate TRD prevalence of 13.6% (Table 1) within MDD. While keeping in mind the often observed caveat that it is difficult to compare TRD prevalence reported by different studies as the definition of TRD used across studies is not consistent [14], the estimate of prevalence from this study is smaller but still consistent with prevalence estimates that ranged from 10–29% as reported in the literature [8], [15], [16]. The smaller prevalence in this study can be explained by the methodological approach to restrict to new episodes of MDD, the requirement of pharmacological treatment and the use of a commercial claims database that represents on average a wealthier and healthier population.

The sizeable TRD prevalence, and associated increased clinical and economic burden, is a cause for concern, and points to a significant unmet need in the options available to treat the disease. While nearly 80% of non-TRD episodes require only one line of treatment, more than 75% of TRD episodes utilize at least four lines of treatment, pointing to considerable changing of therapies in TRD episodes. This is despite the availability of multiple different classes of antidepressants, the extensive use of combination therapy and the frequent changes of regimen. Multiple LOTs, especially later in an episode, can be indicative of treatment failure, as borne out in the findings of the large naturalistic prospective STAR*D trial which showed that an increase in lines of therapy was inversely related to remission rates (remission at Step 1 exit was reported to be 37%, at Step 2 exit 31%, at Step 3 exit 14% and at Step 4 exit 13%) [25]. A related insight from this study is that within an episode the duration of line of treatment was noticeably shorter in TRD episodes than in non-TRD episodes. While the reasons for changing therapies were not contained in the data - these could include lack of efficacy of the current treatment, a pattern of inadequate response in prior MDD episodes which triggers the physician to switch treatment sooner, lack of tolerability or interactions with other medications the patient may be taking - it can be inferred that with the pharmacological options currently available, many patients with depression are unable to achieve and maintain an adequate therapeutic response, leaving them with a need for more efficacious and/or better tolerated treatments.

A significantly greater proportion of TRD patients was found to be afflicted with each of the comorbidities analyzed in the study compared to non-TRD counterparts, showing that inadequately treated depression is associated with an increase in comorbidities. Ivanova et al [7] have reported an increase in osteoarthritis from 4.2% in MDD controls to 5.6% in the TRD-likely cohort, in chronic pain from 14.5% to 23.2% and in fibromyalgia from 3.0% to 6.4%. Greenberg et al [26] have similarly shown higher incidence of comorbidities in TRD-likely employees as compared to MDD but TRD-unlikely employees in each of the 16 categories that they placed comorbidities in, e.g.: comorbidities related to the musculoskeletal system of 53% in TRD-likely employees vs. 33% in MDD but TRD-unlikely employees, 55% vs. 38% in comorbidities related to the respiratory system and 38% vs. 27% in those related to the circulatory system. This greater occurrence of comorbidities in TRD patients adds to the higher utilization of medical resources, which are already inflated on account of longer episode duration, increased number of prescriptions and changes in prescription for TRD episodes, as evidenced by a higher rate of visits for TRD patients (e.g.: 0.06 hospitalizations vs. 0.04 for non-TRD, 7.48 pharmacy claims vs. 5.15, 1.54 office visits vs. 1.04 and 0.87 lab tests vs. 0.71 for non-TRD (Table 7). As a result, the unadjusted cost of hospitalizations, physician visits, pharmacy claims and other outpatient claims are between 2.7 and 5.8 times more in a TRD episode than a non-TRD episode. Results from this analysis are in line with the study by Ivanova et al [7], which reported that average direct 2-year costs were significantly higher for TRD-likely employees ($22,784) compared with MDD controls ($11,733), and that average indirect costs were also higher among TRD-likely employees ($12,765) compared with MDD controls ($6,885). The breakdown of medical resources shown in Table 7 additionally reveals interesting insights related to the contribution of each category of medical resources towards the greater economic burden associated with TRD. For instance, the rate of visits to a psychiatrist in TRD episodes is more than twice that of non-TRD episodes, while the rate of visits to a general physician is only marginally more.

Although a rigorous methodology was designed and executed to carry out this study, the study suffers from the typical limitations associated with any claims data analysis, including coding accuracies and non-availability of clinical and diagnostic information, such as why a patient switched therapies. While detailed business rules were put in place to identify regimens, the identified regimens may not be reflective of the exact regimens being prescribed by the physician or being taken by the patient. Further, the presumption that a change in treatment regimen is effected by a physician only after the previous regimen has been continued for adequate duration and with adequate titration and dosing, may not always be the case in the real world. This is compounded by the lack of information related to compliance of the patient for each prescription drug purchased. Furthermore, failure was based on drug therapy discontinuation or change, but without knowledge of the underlying reason – whether the failure was on account of not achieving remission or not tolerating treatment - owing to unavailability of this information in the database. The methodology of this study utilized a strategy of first identifying new episodes of MDD and from that population characterizing TRD and non-TRD patients and episodes. By imposing the criterion of new MDD episodes, patients with ongoing episodes of MDD who also have or develop TRD are excluded thereby limiting our ability to understand the TRD experience in these patients. Furthermore, this study does not capture mental health services not covered, reported or reimbursed as a claim. Although this analysis of a large claims database may accurately reflect a commercially insured population, it may not be representative of the general population. In fact, those suffering from TRD and unable to work may be less likely to be represented in this database.

## Conclusion

This study confirms that TRD comprises a significant portion of MDD. It analyzes and characterizes the medication utilization patterns within an episode of TRD, including the considerable changing of therapies. This study also highlights the comorbidities and MRU associated with TRD. The consequences of untreated or inadequately treated TRD place significant burden on the patient, payers and society. Therefore, effective alternative therapies for treatment-resistant patients, either as monotherapy or in combination with existing treatment options, will offer great relief to patients suffering from TRD, and may have the added benefit of alleviating some of the comorbidities from which they suffer, resulting in significant medical and economic benefit.
